# MOLGENIS research: advanced bioinformatics data software for non-bioinformaticians

**DOI:** 10.1093/bioinformatics/bty742

**Published:** 2018-08-26

**Authors:** K Joeri van der Velde, Floris Imhann, Bart Charbon, Chao Pang, David van Enckevort, Mariska Slofstra, Ruggero Barbieri, Rudi Alberts, Dennis Hendriksen, Fleur Kelpin, Mark de Haan, Tommy de Boer, Sido Haakma, Connor Stroomberg, Salome Scholtens, Gert-Jan van de Geijn, Eleonora A M Festen, Rinse K Weersma, Morris A Swertz

**Affiliations:** 1 Genomics Coordination Center, University of Groningen and University Medical Center Groningen, Groningen, The Netherlands; 2 Department of Genetics, University of Groningen and University Medical Center Groningen, Groningen, The Netherlands; 3 Department of Gastroenterology and Hepatology, University of Groningen and University Medical Center Groningen, Groningen, The Netherlands

## Abstract

**Motivation:**

The volume and complexity of biological data increases rapidly. Many clinical professionals and biomedical researchers without a bioinformatics background are generating big ’-omics’ data, but do not always have the tools to manage, process or publicly share these data.

**Results:**

Here we present MOLGENIS Research, an open-source web-application to collect, manage, analyze, visualize and share large and complex biomedical datasets, without the need for advanced bioinformatics skills.

**Availability and implementation:**

MOLGENIS Research is freely available (open source software). It can be installed from source code (see http://github.com/molgenis), downloaded as a precompiled WAR file (for your own server), setup inside a Docker container (see http://molgenis.github.io), or requested as a Software-as-a-Service subscription. For a public demo instance and complete installation instructions see http://molgenis.org/research.

## 1 Introduction

In order to improve human health, biomedical scientists are increasingly using large and complex datasets to discover biological mechanisms. Large numbers of patients and control participants are screened with questionnaires, biomedical measurements, high-throughput techniques such as next-generation sequencing of the genome, the transcriptome and the microbiome (Ginsburg, 2014), resulting in large quantities of phenotypic and molecular data (Bowdin *et al.*, 2014). However, many clinical professionals and biomedical researchers do not always have the proper tools to process, manage, analyze, visualize and publicly share these data (Jagadish, 2015) while complying to ‘FAIR’ (Findable, Accessible, Interoperable, and Reusable) (Wilkinson *et al.*, 2016) and ‘ELSI’ (Ethical, Legal and Social Implications) principles.

Several challenges arise when developing software for big data used by biomedical researchers (Raghupathi and Raghupathi, 2014). The first challenge is data capture and data management. Data systems need to be adaptable enough to not only handle today’s data, but also be able seamlessly capture tomorrow’s data formats (Swertz *et al.*, 2010a,b). Current systems are often too strict in terms of importing new data types. As a consequence, systems must sometimes even be taken offline for database redesign (Adamusiak *et al.*, 2012; Swertz *et al.*, 2010a,b). Therefore, a good system needs to allow continuous use while databases can be redesigned and unforeseen data types can be used. The second challenge is to integrate and analyze the data. Biological data is complex and heterogeneous by nature, leading to incompatible data, disorganized systems, and missed opportunities (Auffray *et al.*, 2016; Jagadish, 2015). Data integration solutions are needed to understand the interaction of environmental effects with molecular measurements resulting in certain phenotypes (Stieb *et al.*, 2017), by combining multiple -omics layers (Suravajhala *et al.*, 2016) with clinical data (Higdon *et al.*, 2015). The third and most difficult challenge is to create user interfaces that are easy to understand and interpret the data, but elaborate enough to allow for comprehensive queries, analyses and visualizations needed for biomedical ‘big data’ research.

Here, we present MOLGENIS Research, designed to overcome the aforementioned challenges and follow the natural flow of biomedical research: collect, manage, analyze, visualize and share data.

## 2 Features

MOLGENIS Research is a life science data solution built on top of the MOLGENIS platform. The MOLGENIS platform allows the development of various apps for specific tasks and to upload data models and settings to tailor the platform to a specific use. Below, we present a collection of apps and settings that together form the MOLGENIS solution for Research. These apps are grouped into the five categories that represent the typical flow of research data: (i) Collect: gathering or entering of data into the database; (ii) Manage: inspecting and handling of data inside the database; (iii) Analyze: detect patterns and differences in the data using algorithms and statistical tests; (iv) Visualize: creating graphs and other visualizations; and (v) Share: making data, visualizations, and results available to others.

### 2.1 Collect

MOLGENIS Research offers several ways to enter or upload data into the system. The typical way to add data is to use either the *Single-click Importer* app or the more advanced *Step-by-step Importer* app. Both importer apps accept files in the EMX (Entity Model Extensible) format, and they are well-documented at https://molgenis.gitbooks.io. EMX is a flexible spreadsheet format for tabular data. It allows data modeling at runtime with definitions for each column in a table, meaning the data in the columns are not predefined or locked in place, yet data consistency is checked and preserved. EMX-formats with XLSX-, ZIP-, and TSV-extensions can be uploaded. A more specialized importer accepts VCF and VCF.GZ files for the quick import of genomic data, as well as OWL and OBO formats for importing ontological data. Additional community standard formats can become supported in the future depending on user needs. The *Remote File Ingest* app can access remote servers and securely import data directly over the web. Via the *Questionnaire* app, data can be collected instantly from study participants and imported in the database. Answers filled in by participants are stored directly in the MOLGENIS Research database. Finally, manual data entry can be performed in the *Data Explorer* app by adding rows or columns in database tables.

### 2.2 Manage

**Fig. 1.**
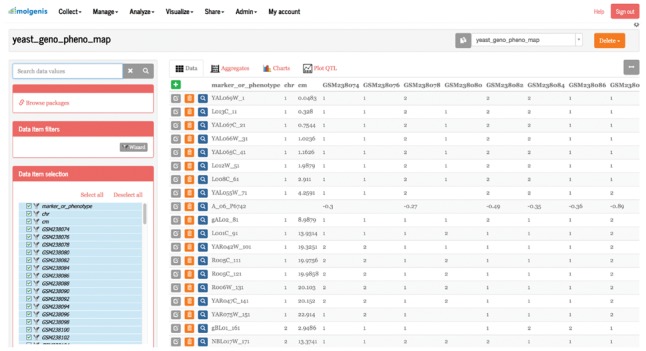
Screenshot of the MOLGENIS Research graphical user interface. Shown here is the *Data Explorer* app, a central place in MOLGENIS Research to enter, enrich, filter, analyze and export datasets

After data collection, MOLGENIS Research has apps to inspect, organize, permit and customize the data. The primary data management app is the *Data Explorer*, which acts as a table viewer. Here, columns can be selected and sorted, and data rows are visible. In addition, datasets can be placed in a hierarchical folder structure via the ‘Package’ system table. See Figure 1 for an impression of the *Data Explorer*. Using the *Navigator* app, datasets can be browsed and viewed in their folder structure. Finally, the *Metadata manager* enables super users to modify the underlying data structure itself to keep up with advancing insights and address unforeseen requirements.

### 2.3 Analyze

MOLGENIS Research enables bioinformaticians to add data analysis tools. For example, the *Data Explorer* is often used to analyze data. Here, the *Filter Wizard* can be used to run queries. In a recent project, the *Descriptive Statistics* app was added to automatically create all descriptive statistics often needed to present in the first table of a manuscript. The *Descriptive Statistics* app automatically recognizes whether data is continuous, binary or categorical, whether it is normally distributed or not and whether there are too many missing values. Based on the outcome it provides, means, medians, counts and percentages.

Use of R, Python and REST APIs allows adding additional data analysis and connections to other data systems. There is a Scripts app in which JavaScript, R and Python scripts can be stored and run by others. These scripts can be written by bioinformaticians, but can be easily run or repeated by researchers without data analysis skills. Using these, specialized tools can be built, [e.g. the GAVIN method (van der Velde *et al.*, 2017)] to automatically classify pathogenicity of genomic variants. Several examples of such add-on analysis tools are showcased in the demo.

### 2.4 Visualize

When genome data is opened, the *Genome Browser* app automatically visualizes genomic loci using the interactive Dalliance genome browser (Down *et al.*, 2011). More scripts are available on the MOLGENIS website and Github repository to generate value distribution plots and consensus in multiple categorical values. Custom reports and visualizations can be added via a templating system (Freemarker) that loads a single row or whole dataset for user-specified formatting rules, and that uses aforementioned scripting capabilities (see Section 2.3).

### 2.5 Share

To support collaborations, MOLGENIS Research has different ways to share and connect the data and to achieve a number of FAIR metrics (Wilkinson *et al*., 2018) such as ensuring identifier uniqueness and persistence, indexing its data tables, offering HTTP access and authorization, and tools to connect data to FAIR vocabularies such as ontologies (Pang *et al.*, 2015a). For joint analysis of datasets, we have developed *Mapping Service* tools to make both columns (Pang *et al.*, 2015a) and values (Pang *et al.*, 2015b) interoperable between datasets so they can be merged. The *Tag Wizard* app can assign meaning to data columns using ontologies, which can be integrated across different datasets using the *Mapping Service* app. Datasets and variables can be made findable without exposing (sensitive) data values by creating a catalogue from a combination of raw data, curated data or interesting results collected in the system. Others can browse this catalogue before contacting or submitting a request for access. MOLGENIS Research supports the complete data access and request workflow designed by the data owner. Super users can also create FAIR endpoints (Wilkinson *et al*., 2017) based on definitions of Metadata, Catalog, Dataset, Distribution and Response, which ensures your data is machine-findable and thereby has increased findability.

## 3 Implementation

MOLGENIS Research is implemented using open and freely usable industry standards. It is available under the GNU Lesser General Public License v3.0 (https://www.gnu.org/licenses/lgpl-3.0.en.html). It is written in Java 1.8 (https://java.com), supported by the Spring MVC framework (https://spring.io). It uses Apache Maven (https://maven.apache.org) to manage dependencies, and runs on an Apache Tomcat (http://tomcat.apache.org) webserver. Data is stored in a PostgreSQL database (https://www.postgresql.org) and indexed by ElasticSearch (https://www.elastic.co) for high performance and horizontal scaling ability by data replication and sharding, respectively. Final storage and query performance depends on specific hardware and software configuration. Its graphical user interface is composed of Bootstrap (https://getbootstrap.com), Vue (https://vuejs.org) and Freemarker templates (https://freemarker.apache.org). FAIR endpoints are implemented in W3C RDF 1.1 Turtle (https://www.w3.org/TR/turtle).

## 4 Conclusion

We have built MOLGENIS Research, a web application for the biomedical field to work with multi-omics datasets without being dependent on bioinformaticians. MOLGENIS Research enables researchers to more efficiently collect, manage, analyze, visualize and share data, as well as offering support to make data FAIR in a flexible and safe way. MOLGENIS Research offers all the advantages of a true database system with detailed data management and access control options, while at the same time being able to grow ‘organically’ by allowing data to be dynamically shaped based on what is needed in practice, and adding custom extensions such as visualizations and algorithms into a running system without downtime. It can be used as a project database from day one as there is no need to design a data model upfront.

Currently, MOLGENIS Research has been adopted by several research projects, including 1000IBD, 500FG and LifeLines. The 1000IBD database (http://1000ibd.org) contains a range of clinical and research phenotypes for up to 2000 patients per -omics type, which includes quantifications of 12 000+ microbiome OTUs, 400+ immunochip markers, and ∼300 RNA-seq experiments. The 500FG database (https://hfgp.bbmri.nl) contains microbiome, metabolomics, cytokine, QTL, cell staining, serum Ig and flow cytometry data for around 500 individuals. Identifier codes for individuals serve as foreign keys that can link data tables together for data integration and analysis. Lastly, the LifeLines data catalogue (https://catalogue.lifelines.nl) contains the metadata for around 40 000 data items available for researchers such as questionnaires, measurements and (blood and urine) sample analyses from a longitudinal study of 167 000 individuals. We expect more projects to follow soon, and gladly invite everyone to help us in expanding and evolving the MOLGENIS Research solution to serve all popular research needs. We strongly encourage interested users to try the demo, download and install MOLGENIS Research at http://molgenis.org/research.
